# Economic Burden of Thalassemia Major in Iran, 2015 

**Published:** 2016-08-24

**Authors:** Firooz Esmaeilzadeh, Azita Azarkeivan, Sara Emamgholipour, Ali Akbari Sari, Mehdi Yaseri, Batoul Ahmadi, Mohtasham Ghaffari

**Affiliations:** ^a^ Department of Health Management & Economics, School of Public Health, Tehran University of Medical Sciences, Tehran, Iran; ^b^ Pediatric Hematology Oncology, Transfusion Research Center, High Institute for Research and Education in Transfusion Medicine, Department of Thalassemia Clinic, Tehran, Iran; ^c^ Pediatric Congenital Hematologic Disorders Research Center, Shahid Beheshti University of Medical Sciences, Tehran, Iran; ^d^ Department of Epidemiology and Biostatistics, School of Public Health, Tehran University of Medical Sciences, Tehran, Iran; ^e^ Environmental and Occupational Hazards Control Research Center, School of Public Health, Shahid Beheshti University of Medical Sciences, Tehran, Iran

**Keywords:** Cost of Illness, beta-Thalassemia Major, Health Care Costs, Iran

## Abstract

**Background:** Major Thalassemia is an autosomal recessive disease with complications, mortality and serious pathology. Today, the life expectancy of patients with major thalassemia has increased along with therapeutic advances. Therefore, they need lifelong care, and caring for them would incur many costs. Being aware of the patients’ costs can be effective for controlling and managing the costs and providing efficient treatments for the care of patients. Hence, this study was conducted to estimate the economic burden of the patients with major thalassemia.

**Methods:** Totally, 198 patients with major thalassemia were randomly selected from among the patients with major thalassemia in Tehran, Iran in 2015. The economic burden of the patients was estimated from a social perspective and through a bottom-up, prevalence-based approach.

**Results:** The average annual cost per patient was estimated $ 8321.8 regardless of the cost of lost welfare. Of this amount, $ 7286.8 was related to direct medical costs, $ 461.4 to direct nonmedical costs, and $ 573.5 to indirect costs. In addition, the annual cost per patient was estimated $ 1360.5 due to the distress caused by the disease

**Conclusions:** Considering the high costs of the treatment of patients with major thalassemia, adopting new policies to reduce the costs that patients have to pay seems necessary. In addition, making new decisions regarding thalassemia screening, even with higher costs than the usual screening costs, can be useful since the costs of treatment are high.

## Introduction

Thalassemia major is an autosomal recessive disease with serious complications, mortality and pathology^1,2^. A higher prevalence of thalassemia is mainly seen in developing countries ranging from the Mediterranean Sea, including Turkey, Iran, and India to South East of Asia including Thailand and southern China^3,4^.

Due to the high costs of treatment and the lack of receiving adequate measures, many thalassemic children and adolescents die in poor countries^5-7^. Timely blood transfusion appears to prevent early symptoms of the disease, and the patients will continues to grow up^8,9^. In many cases, however, transfusion reactions such as excess iron is deposited in the body, causing heart failure^10,11^, chronic liver diseases, endocrine problems, growth disorders, osteoporosis, etc., leading to mortality in these patients^12,13^. Thus, health management as well as planning the required services for early diagnosis and treatment of these patients seems essential ^14,15^.

Today, the life expectancy of patients with major thalassemia has significantly increased along with therapeutic advances^16^, and this has changed thalassemia from a fatal to a chronic disease^17,18^. As a result, thalassemic patients need lifelong care^19^, but it requires high costs including the costs of blood transfusion, iron chelation drugs, laboratory tests, treatment of side effects, periodic visits, and indirect costs such as the costs of lost opportunities as well as lost welfare and quality of life^6,20^. Since health care funders are seeking to control the costs and effectively allocate the resources^20^, having knowledge of the invested costs for thalassemic patients is essential for optimal allocation of resources in this sector. Some studies in the United Kingdom^21^, Thailand^22^, Taiwan^23^, Iran^20^ and Israel^24^, were conducted to estimate the costs of thalassemia treatment, but the estimated annual costs to treat a thalassemic patient were reported quite differently in those studies so that the costs had been estimated $ 950 in Thailand and $ 39472 in Israel.

Due to the changes in medications and the survival of thalassemic patients, the costs in this sector have changed significantly compared to previous years. Therefore, the aim of this study was to estimate comprehensively the costs and economic burden of the patients with major thalassemia. Results of this study can be effective to control, manage the costs, and provide efficient treatments that are taking place in different sectors to care for thalassemic patients. Furthermore, being aware of the treatment costs of the patients with major thalassemia can help making decisions for screening programs.

## Methods

The year 2015 was considered as the base year for cost estimation, and the economic burden of thalassemia was assessed from a social perspective, with a bottom-up method based on a prevalence-based approach. In this regard, the total costs included all direct, indirect and intangible costs spent for thalassemic patients within a one-year period ^22^. To estimate the average cost per thalassemic patient, it was necessary to review the patients' files, and all the services provided had to be included. Hence, patients with thalassemia were sampled.

The number of samples was estimated to be at least 170 people on the basis of Altman formula ^22^ (where the sample size for each independent variable is said to be at least ten), the number and grouping of independent variables (age (under 10 years old, 11 to 20 years old, 21 to 30 years old, 31 to 40 years old and above 40 years old), sex(male, female), blood transfusion pattern (never, occasionally , often), splenectomy (yes , no), distance from home to service-providing center, chelation treatment (oral , injection ), blood type (washed, unwashed ) ).

The study was confirmed by the Ethics Committee of Tehran and Shahid Beheshti Universities of Medical Science, Tehran, Iran.

To do the sampling, 200 people were selected through the simple random selection method and using the random number table among the active cases available in Tehran Association of Supporting Thalassemic Patients (It should be noted that almost all patients with major thalassemia in Tehran are members of Tehran Association of Supporting Thalassemic Patients). After providing the necessary information, 198 patients with major thalassemia enrolled the study willingly, (two patients stated that the previous studies conducted on them had had no effect on their welfare. So, they refused to participate in the study).

### Calculation of direct costs

direct medical costs including transfusion, iron chelation therapy, medications, tests, doctors’ visits, and hospitalization costs were extracted by the review of patients’ records, interviews with patients or one of their family members, interviews with the doctors who visited the patients continuously, and review of the patients’ records, and they were calculated by adding the subsidies of the Ministry of Health as well as insurance payments as service buying. Since blood transfusions and other types of caring for thalassemic patients are free in health centers of Iran, the salaries and benefits of the personnel of these centers as well as the costs of the transportations done in the health centers were assigned to each patient based on $c_{i}=C_{I}\frac{b_{i}}{B_{I}}$ formula and were added to the costs calculated for each patient in the previous stage.

c_i_‏ = Costs related to the patients i‏.

C_I_ = the total costs allocated to center *I* where the patient i has been selected from

b_i_= the number of blood bags that the patient i has received over a year

B_I_= the total number of blood bags injected to the patients over a year in *I* centers

### Calculation of indirect costs

To estimate the indirect costs, a notebook was given to each patient or their parents, and after training them what to do, the patients were asked to write down the time cost as well as other costs associated with the disease, including the time of absence from work, the time spent on going to health care providers, the time spent by family members to take care of the patients or pay a home nurse to do so, the supports and decoration changes necessary for the patients to be able to cope with the disease, transportation and others costs. The patients were followed by phone for two months (they were called once a week and their probable questions were answered as they were reminded to write down in their notebooks). After two months, the notebooks were collected, and according to the formula $I_{i}=GDP_{pr}=\frac{t_{i}}{8*289}$, the lost time changed into the lost income and the indirect costs for each patient were calculated by adding the other registered costs.

t_i_: lost time because of the thalassemia in patient i,

GDP _pr_: Gross domestic product per capita.

The annual costs of capital equipment’s in the service providing centers were calculated based on the value of capital goods, equipment depreciated value and useful life of the equipment. In addition, the costs of water, electricity, telephone, gas and other utilities were estimated according to the past-year bills as well as warehousing records. The building costs were also included as the annual rental costs. All costs were firstly allocated to the service providing centers based on the allocation criteria, and were then allocated to the patients based on the allocation criterion stated in the direct costs section. Using the disability weight caused by thalassemia, the lost welfare costs due to the complications of the disease as well as the number of YLDs resulted from thalassemia were calculated and multiplied by GDP to estimate the per capita of some part of the lost welfare costs for each patient.

## Results

Totally, 102 of participants (51.5 %) were male. The youngest and the oldest patients in the study were two and 45 years old, respectively, and the average ages of male and female participants were respectively 23.3 ± 9.7 and 25.8 ± 9.9 yr. On average, the patients had started receiving blood at the age of 20.9 ± 18.1 months and had referred to the centers 5.2 ± 19.1 times a year to receive blood. The average annual blood received by each patient was 10.2 ± 30.8 units. Thirty seven percent of the patients had referred for services with a family member. Of course, being accompanied by somebody varied among patients of different age groups so that all patients in the age group below 10 years were accompanied by somebody, and most patients older than 40 years had referred alone (Table 1).

**Table 1:** Average annual services received by thalassemic patients per patient

**Age groups yr)**	**No. of bloodletting (times)**	**Received blood count (bags)**	**Washed blood (bags)**	**No. of daily accompaniers (Person)**	**Deferoxamine**	**Deferasirox**	**Deferiprone**
0-10	17.45	20.04	1.98	19.19	338.8	562.7	84.7
11-20	18.53	27.08	3.00	12.49	599.6	486.2	586.8
21-30	18.78	30.33	3.74	3.83	786.6	430.6	828.9
31-40	20.33	26.11	3.90	3.88	891.4	291.4	1114.3
>40	19.62	20.42	3.26	1.90	925.7	72.0	0.0
Total	19.01	26.79	3.40	7.54	725.2	404.9	717.8

Table 2 shows the average direct and indirect costs associated with thalassemia as a breakdown of age groups. Among the expenses related to the disease, the highest and the lowest costs had been allocated to the medications taken by the patients (60.4%) and splenectomy in thalassemic patients (0.54%), respectively. Except the costs related to the lost opportunities of the families as the highest expenses in the age group 0 to 10 years, this age group had the least expenses in terms of other items. The average total costs of thalassemic patients increased with age and reached its highest level in the age group 21 to 30 years. After that, the costs per patient reduced again in the next age groups.

**Table 2:** Average annual costs per thalassemic patient ($)

** Type of costs**	**Age groups (yr) **					
	**0-10 **	** 11-20**	** 21-30 **	** 31-40 **	** gt;40 **	**Total **
Blood	836.2	1,130.0	1265.6	1089.5	852.1	1118.0
Medical Visits	170.6	178.9	180.9	192.9	191.9	182.9
Nursing Services	219.3	307.1	345.8	298.4	222.7	303.7
Laboratory Services	121.8	143.2	131.5	138.2	166.2	136.6
Diagnostic Services	85.8	177.3	244.4	261.4	269.7	216.0
Medicine	3764.5	4806.5	5394.4	5404.7	4508.5	5026.4
Deferoxamine pump and other consumer items in home	143.2	150.4	155.2	160.0	177.1	155.0
Hospitalization	50.1	19.0	147.5	153.6	58.4	103.4
Splenectomy	44.8	44.8	44.8	44.8	44.8	44.8
Going to the service providing centers	189.3	190.3	215.6	216.3 184.3	205.3
Transportation	64.1	68.1	69.0	74.7	72.1	69.8
Lost opportunities for patients	No data ^a^	293.9	429.0	457.9	448.2	356.2
Lost opportunities for patients’ families	436.7	310.5	147.5	148.4	111.1	217.4
Building rent and other costs related to buildings	139.3	188.3	209.8	183.4	141.1	186.3
Lost welfare	No data ^b^	No data ^b^	No data ^b^	No data ^b^	No data ^b^	No data ^b^
Total	6265.8	8008.3	8981.0	8824.3	7448.1	8321.8

^a^ In 2015, samples of patients under 15 years had lost 810 working days to get the services. Given that this age group was not included in the working age, changing it to monetary costs was refused. ^b^ According to Naghavi ^25^, Years Lost due to Disability associated with thalassemia is 25 %. Given the per capita GDP (Gross Domestic Product) of $ 5442 for Iran in 2014 ^26^ it can be said that, in addition to the costs expressed in Table 2, almost all patients lose $1360.5 a year for costs associated with pain and suffering caused by the disease.

Regarding the costs of the drugs taken by thalassemia patients, iron chelation drugs accounted for 90.7% of the drug costs. Among the iron chelation drugs, deferoxamine accounted for 57.5% of the total costs of drugs and 63.4% of the costs related to iron chelation ones. Among the non-chelation drugs, the most expensive ones were osteoporosis drugs so that they included 4.4% of the total costs of drugs and 47.8% of non- chelation drug costs (Table 3). In terms of the number consumption, iron chelation drugs were the most frequently used ones, and vitamins, folic acid and aspirin were placed in the next positions, respectively.

**Table 3:** Annual costs of medications taken by thalassemic patients (n=198)

**Drugs**	**The cost per patient ($)**	** The total cost ($)**
Deferoxamine	2892.5	572,722.1
Deferasirox	1550.6	307,026.0
Hydroxyurea	1.2	244.6
Deferiprone	115.0	22,774.7
Osteoporosis drugs	223.4	44,239.4
vitamins	73.0	14,450.1
Folic acid	2.5	497.6
Insulin	19.0	3753.9
Hormonal drugs	111.3	22,043.9
Interferon	18.2	3605.3
Ribavirin	0.7	134.6
Antibodies	3.9	770.8
Furosemide	5.2	1024.0
Splenectomy Vaccines	7.8	1547.8
Clotting agents	2.0	386.8
Total	5026.4	995,221.6

**Figure 1:**
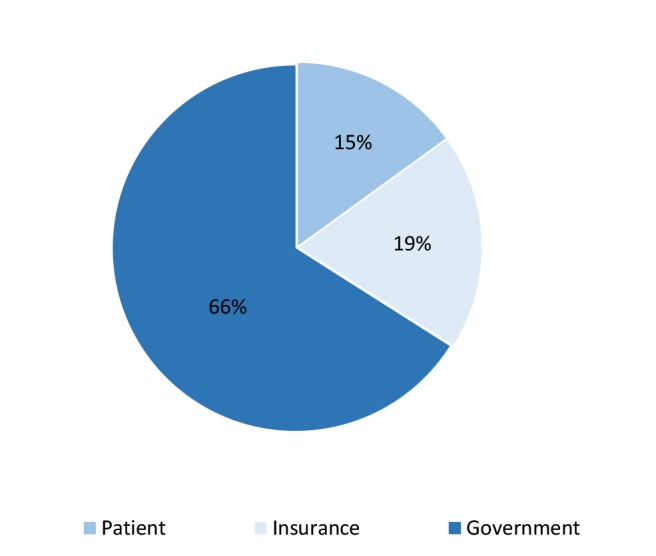
The share of the government, insurance companies and patients in thalassemia-related costs

Totally, $ 1,647,722.6 had been spent for198 thalassemic patients in 2015 and the the average‏ cost per patient, regardless of the lost welfare costs, was estimated as $8321.8. Of this amount, $7286.8 related to direct medical costs, $461.4 to direct non-medical costs and $573.5 to indirect costs. In addition, the lost welfare costs for all patients (n = 198) were estimated to be $269,379. According to Figure 1, the greatest part of the disease costs was paying by the state and from public resources. However, every patient under study had spent an average of $1218.8 to receive the services regardless of the lost welfare costs, and the annual contribution of insurances to treatment costs was estimated as $1607.8 per patient.

Results of the studies show that currently the treatment of patients with thalassemia major is very costly^21^. In the present study, the annual cost of treating a thalassemic patient, regardless of the lost welfare costs, was estimated $ 8321.8 due to the disease complications and the lost opportunity costs caused by premature death. Given that there are approximately 18,000 patients with major thalassemia in Iran, it can be said that thalassemia imposes $ 149,792,964.3 a year to the Iranian health system.

A study carried out in Taiwan ^23^ had estimated the annual costs of thalassemic patients as $ 7464.4 similar to our results. The studies done in Iran ^20^ and Thailand ^22^ have estimated the annual costs of each thalassemic patient as $ 2252.7 and $ 950, respectively. The low costs in the mentioned studies are justifiable with the low doses of iron chelation drugs and administered blood units (hence, the low cost of lost opportunities, the costs of going to the centers, etc.) so that in the studies mentioned above, each patient had received an average of 33 and 40 vials deferoxamine during a year, respectively, while in the present study each patient received an average of 725 vials deferoxamine a year in addition to other iron chelation drugs. Taking into consideration the life expectancy of 50 years for thalassemic patients ^24^, the annual cost of the lost opportunity due to premature death in these patients will be equal to $ 2558.1. By adding the lost welfare costs as well as the direct and indirect treatment costs, the annual and the lifetime expenses of a thalassemic patient (without discount fees) were estimated $ 12,240.4 and $612,020.0, respectively. In the United Kingdom ^21^, the lifetime expenses of a thalassemic patient were estimated $ 898,851 without discount fees. Other studies in Israel ^24^ had calculated the lifetime expenses of a thalassemic patient (with a life expectancy of 50 years) and their annual costs as $1,971,380 and $39,472 respectively.

A study in America estimated the annual treatment costs of thalassemic patients who had moderate and severe diseases with a combination of deferropiron and deferrioxamin as 22199 and 55690 dollars, respectively, and 22404 to 53095 dollars for treatment with defferoziroxen ,^27^. In those studies, the costs were almost twice as much as the ones estimated in this study. Since the list of the expenses was not separately available in the above-mentioned studies, we cannot certainty determine the differences in the costs, but high expenses in those countries can be associated to the high cost of lost opportunities in there due to the high GDP per capita and high wages.

In the present study, the average treatment costs increased with age, and this increase seems reasonable with the increase of required services for thalassemic patients. However, after the age group 30-20 years, the costs reduced again contradicted to the increasing need of the patients to services as their ages increased. The reason could be that some patients whose disease was more severe and needed more services died in the age groups 20-30 yr or the first years of 31-40. In general, the relatively healthier patients who had relatively lower costs were entered the age group 31-40 years and this trend reduced the costs in the next age group, too. Because the blood is free for these patients and the coverage of iron chelation drugs is fairly complete, the patients receive these services almost entirely. However, they pay about 15% of the costs regardless of the costs of pain and distress as well as the premature death caused by the disease. This can be unaffordable for many patients and can bring about the costs that might lead to poverty, so that some patients unable to pay the costs do not receive supportive treatments such as vitamins and osteoporosis medications. Therefore, the following policies should be taken into consideration by the related policy makers: the full insurance coverage of thalassemia medications, giving subsidies to the patients, and providing supportive drugs by the thalassemia associations.

This study has considered all aspects of costs comprehensively and from a social perspective, and it has been tried to keep track of the patients studied so that all costs associated with the disease would be carefully included. Hence, it can be very useful in making decisions for effective allocation of the resources. However, life expectancy considered for these patients may be somewhat different from the life expectancy of thalassemic patients in Iran, and this can affect the costs associated to the life expectancy of thalassemic patients.

## Conclusions

According to the results of this study and due to the high costs of treatment of patients with thalassemia major, it seems necessary to adopt new policies in short term including full insurance coverage and giving subsidies to the patients in order to reduce the costs that the patients have to pay. Furthermore, some studies have described bone marrow transplantation to be very frugal‏ ^28^, which can be considered in the long time to reduce the costs in this sector. In addition, due to the low costs of thalassemia screening ^24^, and high costs of treatments, new decisions have to be made in relation to doing thalassemia screening even with higher costs than the usual ones. Active screening as well as making it free in areas with a high prevalence of minor thalassemia where the residents have low income seems to be effective.

## Acknowledgments

This is an original study of a Ph.D. thesis and an academic research supported by Tehran University of Medical Sciences (TUMS) (Grant No: 94-01-27-25780). The authors appreciate the collaboration of Tehran University of Medical Sciences, Iran University of Medical Sciences, Shahid Beheshti University of Medical Sciences and Zafar Thalassemia Clinic.

## Conflict of interest statement

The authors have no competing interests.

## Highlights

- Thalassemia treatment costs are very high and much of the costs are related to the drugs received by the patients.
- Most of the costs of drugs are related to iron chelation and Deferoxamine drugs.
- The insurance coverage of the services received by thalassemia patients is relatively low.
- Patients with thalassemia major pay a lot of money to receive the services and this can lead to catastrophic expenditures for them.
